# Coronary CT and the Coronary Calcium Score, the Future of ED Risk Stratification?

**DOI:** 10.2174/157340312801784989

**Published:** 2012-05

**Authors:** Leticia Fernandez-Friera, Ana Garcia-Alvarez, Gabriela Guzman, Mario J Garcia

**Affiliations:** a Departamento de Cardiologia, Hospital Universitario Marqués de Valdecilla, Santander. Spain; b Centro Nacional de Investigaciones Cardiovasculares (CNIC), Madrid. Spain; c Thorax Institute Cardiology Department, Hospital Clinic, Barcelona, Spain; d Hospital La Paz, Madrid. Spain; e Montefiore Heart Center-Albert Einstein School of Medicine. New York

**Keywords:** Emergency department, acute chest pain, coronary artery calcium score, coronary computed tomography.

## Abstract

Accurate and efficient evaluation of acute chest pain remains clinically challenging because traditional diagnostic modalities have many limitations. Recent improvement in non-invasive imaging technologies could potentially improve both diagnostic efficiency and clinical outcomes of patients with acute chest pain while reducing unnecessary hospitalizations. However, there is still controversy regarding much of the evidence for these technologies. This article reviews the role of coronary artery calcium score and the coronary computed tomography in the assessment of individual coronary risk and their usefulness in the emergency department in facilitating appropriate disposition decisions. The evidence base and clinical applications for both techniques are also described, together with cost- effectiveness and radiation exposure considerations.

## BACKGROUND

Acute chest pain presents an expensive and high risk challenge for the global healthcare system. More than 6 million patients with chest pain present to the emergency department (ED) in the United States each year [1]. Early and accurate triage of these patients is crucial to both treatment and prognosis but remains clinically challenging. The limited predictive value of clinical history and physical examination complicates accurate risk stratification, particularly in patients with normal cardiac biomarkers and non-diagnostic electrocardiograms (ECG) [2]. As a consequence, over 60% of patients admitted to the hospital for evaluation of acute coronary syndrome (ACS) are discharged with a non-cardiac diagnosis [3], resulting in unnecessary use of medical resources. Conversely, the rate of missed diagnosis of ACS in the ED remains unacceptably high, ranging from 2% to 8%, with missed diagnoses associated with a two-fold increase in mortality [4]. The financial cost associated with the diagnostic ambiguity of undifferentiated chest pain is enormous, estimated at $10 billion annually in the United States [5] . 

Although many routine diagnostic modalities are available in the ED for the evaluation of undifferentiated chest pain, none has adequate sensitivity to effectively rule out the diagnosis of myocardial ischemia. The standard 12- lead electrocardiogram lacks adequate sensitivity and negative predictive value (NPV) to rule out any form of ACS [6]. Troponin measurement, considered the diagnostic gold standard for myocardial infarction, has a low sensitivity to exclude myocardial ischemia or early manifestations of ACS. Rest echocardiography has limited sensitivity to detect ACS, especially in cases of unstable angina, and false negative results are frequent [7]. Although the additional use of stress echocardiography improves the negative and positive predictive values, it requires highly experienced sonographers and physician readers and, as a result, is not universally available [8]. Exercise treadmill electrocardiography is limited by a moderate predictive accuracy to detect coronary artery disease (CAD) and the frequent presence baseline ECG abnormalities (e.g., left-bundle-branch block) that preclude accurate interpretation [9]. Single-photon emission computed tomography (SPECT) demonstrates excellent sensitivity (>90%) and good specificity (67% to 78%) when compared with coronary angiography [10] and moreover, it has been shown to have prognostic value for predicting the short- and long-term risk of cardiac events [11]. However, this technique is not ideal for initial ED evaluation of ACS because it is relatively costly, time-consuming and requires significant radiation exposure. More recently, shortage of radiolabeled technetium compounds has resulted in increased utilization of Thallium-201, further increasing patient radiation exposure. Overall, available functional tests are generally limited in their suitability for ED triage of undifferentiated chest pain because of the need of specifically trained personnel, prior exclusion of acute infarction through serial cardiac biomarkers measurements and the high frequency of non-diagnostic results. 

## CURRENT APPROACHES FOR GLOBAL CARDIOVASCULAR RISK STRATIFICATION

In an effort to rationally triage patients with acute chest pain, appropriate risk assessment should be utilized in the ED. Although multiple CAD related risk stratification tools exist, their applicability to the ED is limited. The most common clinical decision instrument utilized in the ambulatory care setting is the Framingham multivariable score system (FRS). This score, which is comprised of multiple clinical variables, is used to predict the 10 year risk of coronary heart disease (including coronary death, myocardial infarction and angina) [12]. The risk of suffering an adverse cardiac event is classified as low, moderate or high, when the predicted risk is less than 10%, between 10% and 20% or greater than 20% respectively [13]. Although useful for the longitudinal long term management of patients in the ambulatory setting, the sensitivity and negative predictive value provided by the FRS is not adequate to meaningly inform disposition decisions in the ED. (PROCAM score) [14, 15].

Similarly, risk scales have been developed to stratify individual short term cardiovascular risk according to clinical presentation, ECG and cardiac biomarkers. One widely used score is the thrombolysis in myocardial infarction (TIMI) risk score (Table **1**). This seven point scale classifies the target population into 3 groups based on their 14 day risk of adverse cardiac events: high risk patients (TIMI risk 5-7), who are commonly referred for urgent coronary angiography, intermediate (TIMI score 3-4) and low risk (TIMI score 0-2) patients who generally undergo a period of observation and serial biomarker testing followed by a risk stratification study of some type [16]. To help facilitate this process, so called chest pain observation units have become commonplace in many tertiary care centers. These units typically provide a standardized approach to the evaluation of acute chest pain that combines information from serial ECG, cardiac biomarkers, and stress testing, allowing an expedited, protocoled cardiac evaluation without the time or expense of an in-patient stay. 

## CORONARY CALCIUM SCORE IN THE EVALUATION OF ACUTE CHEST PAIN IN THE ED

The CAC score has been proposed as an alternate approach for stratification of global cardiac risk, evaluation of chest pain patients and prediction of future cardiac events. Electron-beam computed tomography (EBCT) and CCT are the primary imaging modalities for CAC quantification. Both technologies employ thin axial slice computed tomographic imaging. While EBCT moves the X-ray source point electrically providing faster acquisitions, the CCT has higher spatial resolution and lower hardware costs. Coronary artery calcification is defined based on X-ray attenuation as hyperattenuating lesions >130 Hounsfield Units. The most widely used and established method to quantify CAC is the Agatson score which is determined by calcified lesion area and a calcium density factor [17]. Other measurements, such as the calcium volume score and calcium mass or more recently, lesion and vessel-specific CAC score, are not routinely used in clinical practice. However, the calcium volume score seems to better reflect increases in plaque size and reduce variability between scans [18].The total score corresponds to the sum of all lesions in all three coronary arteries, and is commonly expressed in age and gender specific threshold values to improve diagnostic accuracy.

### Evidence Base: CAC Quantification and Relation with Cardiovascular Events

CAC is a quantifiable marker of atherosclerotic disease [17] which correlates well with histological, intracoronary ultrasound and angiographic measures of coronary plaque burden [19, 20]. Furthermore, CAC score is an established predictor of cardiovascular events [21- 23]. However, the role of CAC score as part of the initial evaluation of patients in the acute setting remains controversial. 

Over a decade ago, McLaughlin *et al*. [24] demonstrated the usefulness of CAC score in this scenario when they evaluated 134 patients with acute chest pain and a normal or non-diagnostic ECG. The prevalence of the presence of CAC in this study was 64%. The authors concluded that patients with CAC score of 0 could be safely discharged based on a demonstrated NPV of 98%. Similarly, Laudon *et al*. [25] suggested that no further testing was needed in the cases of zero CAC score after studying 105 patients with acute chest pain, normal biomarkers and non-diagnostic ECG’s with several testing modalities, including EBCT scanning, exercise ECG testing, conventional coronary angiography, radionuclide stress testing and echocardiography. Accordingly, Sarwar *et al*. [26], based on a NPV of 93% from a systematic review of 18 studies, argued that patients with a CAC score of 0 are highly unlikely to have CAD and do not need further testing. More recently, Fernandez-Friera *et al* [27] found only 2 patients (1.5%) with significant CAD among 133 patients with zero CAC score evaluated with CCT in the ED setting. Overall, the sensitivity of a zero CAC score to predict abnormal cardiac testing in these series was high (97-100%); while the specificity was only moderate (40-63%) (Table **2**). 

The use of CAC score has been also demonstrated to predict cardiovascular events. Georgiou *et al*. [28], prospectively evaluated 192 patients with acute chest pain at a mean follow-up of 4.2 years, and observed higher annual event rate among subjects with high CAC score. Nabi *et al*. [29] reported an excellent short-term outcome for patients with acute chest pain and CAC score of 0. In the Rule Out Myocardial Infarction Using Computer Assisted Tomography (ROMICAT), only 1 of 197 patients with CAC score of 0 had a cardiac event during 6-month follow up [30]. Laudon *et al*. [31] studied 263 low-intermediate risk chest pain patients, and concluded that in the absence of CAC, myocardial ischemia is very unlikely and long-term (5-year) prognosis is excellent, with no primary or secondary cardiac outcomes occurring in the study group during this time period.

The are however, other published studies that suggest less diagnostic utility. For example, Rubinstein *et al*. [32] found a high incidence of significant CAD among patients without CAC (7%) and with low CAC score (17%). In another study of high risk patients, 5 out of 13 (39%) patients with a CAC score of 0 had significant CAD. It should be noted however that the overall prevalence of significant CAD was nearly 70% in this study [33]. The Gottlieb group [34] observed that 12% of vessels without CAC had significant CAD in patients referred for elective coronary angiography. Of clinical importance, each of these studies was performed in a clinical setting with a remarkably high prevalence of significant CAD (56%-70%) and subsequently, the NPV afforded by a CAC score was lower. As with any test, it is necessary to define the study population and its prevalence of disease to accurately assess the utility of CAC score.

More recently, large multi-center studies have reported the use of CAC score for diagnosis of obstructive CAD in symptomatic patients. The overall sensitivity and specificity for CAC to predict obstructive CAD on invasive angiography was 95% and 66% respectively [35, 36]. Furthermore, when CAC score is greater than 100, the sensitivity decreased to 87% and the specificity increased to 79% [37], and when CAC score is less than 100, there is low probability (less than 3%) of abnormal perfusion defect on nuclear stress test [38] or significant CAD on cardiac catheterization [35]. However, there is some concern that these studies are subject to verification bias, which could raise the sensitivity and lower the specificity. 

CAC scoring has been reported to improve diagnostic discrimination over conventional risk factors in the identification of persons with angiographic CAD [37] and to offer potential benefit when combining with other imaging modalities [39]. Shavelle [40] studied 97 patients who underwent technetium-stress, treadmill-ECG testing, and CAC score and observed that the combination of a positive CAC score and abnormal treadmill-ECG raised the specificity for obstructive CAD. Another potential use of CAC scoring is to determinate the etiology of the cardiomyopathy. It has been shown in 120 patients suffering from heart failure of unknown etiology, that the presence of CAC was associated with 99% sensitivity for ischemic cardiomyopathy [41].

In summary, the high sensitivity (95% to 99%) and NPV (96-100%), as well as the low long-term follow-up event rates of a zero CAC score may improve accurate and efficient ED evaluation of undifferentiated chest pain without the traditional requirement of a prolonged observation period or costly advanced imaging studies. In this way, exclusion of CAC may act as an effective filter to determine which patients warrant more advanced diagnostic testing or hospital admission, especially among patients with normal cardiac biomarkers and non-diagnostic ECG’s. 

### Clinical Application of CAC Score in the ED for Chest Pain Evaluation: Selection of Patients

Currently, there are no published guidelines for the use of CAC score in the assessment of acute chest pain in the ED; however, it seems reasonable to extrapolate data from Appropriate Use Criteria for CCT of several American Societies [42, 43], from the CCT consensus statement of the North American Society of Cardiac Imaging and The European Society of Cardiac Radiology on acute chest pain [44] and from the ACC/AHA 2007 Clinical Expert Consensus Document on CAC scoring [45]. 

The appropriate use criteria of CCT have been reported to help clinicians with proper patient selection criteria and avoid overuse. By these recommendations, the use of the CCT and moreover, CAC score, should be restricted to patients at low to intermediate cardiac risk (overall TIMI score ≤ 4) whose first set of cardiac biomarkers and initial ECG results show no sign of acute myocardial ischemia. Similarly, The American Heart Association suggested that the use of CAC score might be recommended in intermediate risk patients to improve cardiovascular risk assessment (Class IIb) as well as in chest pain patients with equivocal or normal electrocardiogram and negative cardiac biomarkers. For patients with established CAD, CAC has poor specificity in the acute setting, and therefore, diagnostic and prognostic value in this population thus remains controversial, limiting its widespread use. As opposed to CCT, atrial fibrillation, obesity or renal insufficiency are not exclusion criteria to performing CAC scoring because there is no need for contrast agents and the post-processing analysis is not limited by the presence of arrhythmias. 

### CAC Score in the ED: Advantages and Limitations

CAC scoring appears to provide valuable information for initial risk stratification of the ED chest pain patient. Some advantages include its non-invasive nature with a NPV that is similar to stress testing. Unlike other functional studies, it is not limited by concurrent medications, the patient’s ability to exercise, or baseline wall motion or ECG abnormalities. Additionally, there is no need for iodinated contrast, specific patient preparation or cooperation. CAC scoring is inexpensive, faster, easier to perform and more available than other imaging techniques with a low radiation dose. Another potential advantage over functional studies is the ability to detect coronary calcium in non-obstructive lesions, providing the opportunity to initiate medical therapy in the early stages of non-obstructive atherosclerotic disease that by definition would not be identified by traditional stress testing. 

The most important limitation of the CAC score is that presence of CAC does not provide evidence of an ACS. Consequently, a non-zero CACS must be viewed as a non-diagnostic study and therefore should be followed by additional diagnostic imaging. Moreover, because the vessel lumen is not visualized this test cannot quantify the severity of the coronary artery stenosis however, high levels of CAC are related to an increased likelihood of obstructive CAD [46]. Another limitation is that the presence and severity of non-calcified plaques cannot be assessed by this examination, missing so called “soft” plaques that are vulnerable to rupture and thrombosis. In addition, because high inter-observer and inter-study variability for CAC evaluation have been described due to different available methods to evaluate CAC, serial CAC quantification cannot be routinely recommended [45]. 

## CORONARY COMPUTED TOMOGRAPHY IN THE ED

### Technical Evolution

The introduction of 16 and 64 multi-detector CCT scanners overcame most of the limitations of early-generation CCT for the evaluation of coronary artery stenosis. Improvement of spatial and temporal resolution, allowing volumetric acquisition of isotropic 0.4-mm voxels with up to 0.33-second gantry rotation time and 165 millisecond temporal resolution, and reduction of scan times to 10 or 20 seconds for scanning the heart or the entire thorax respectively, made feasible the use of CCT in the ED. Introduction of 256 and 320 detector rows and dual source scanners have further improved diagnostic accuracy and feasibility. Dual source and 320 detectors scanners allow complete volume coverage of the heart in a single heartbeat, drastically reducing the susceptibility to arrhythmias and radiation dose. These improvements will likely extend CCT applications in the ED. 

### Evidence Base: Contrast-Enhanced CCT in the ED

**Fig. (1):**
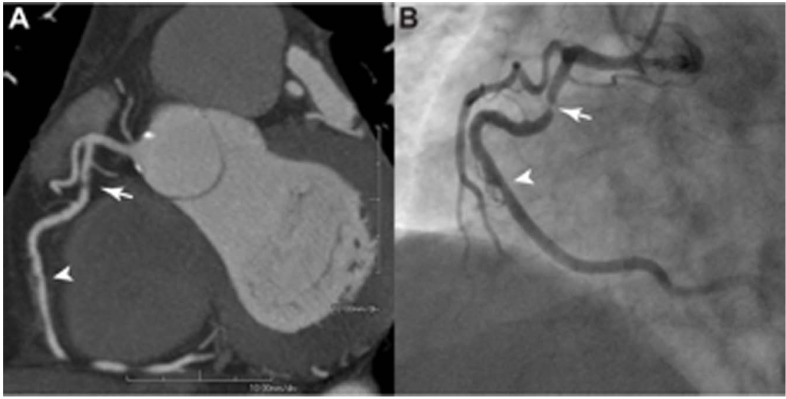
Cardiac computed tomography study and invasive coronary angiography on the same patient. **A**: Multiplanar reformatted image demonstrating a severe stenosis in the proximal right coronary artery (arrow) and mild stenosis in the mid right coronary artery (arrow-head). **B**: Catheterization confirmed the presence of severe and mild stenosis in the proximal and mid right coronary artery, respectively.

The accuracy of CCT for assessing the presence and severity of coronary stenosis compared with invasive angiography has been extensively reported [47] (Fig. **1**). In the acute setting, multiple studies including two meta-analysis, have evaluated the accuracy of CCT in the evaluation of patients presenting with acute chest pain in the ED (Table **3**). One of the first studies assessing this issue, reported by Sato *et al*. [48], used 4 and 16-detectors scanners and found a sensitivity of 95.5% and specificity of 88.9% to detect ACS compared to catheterization as a gold standard. Gallagher *et al*. [49] compared CCT to stress nuclear imaging for the diagnosis of ACS in 85 patients and found that CCT accuracy was at least as good. A meta-analysis including 9 studies totaling 566 patients using scanners with 64 or fewer detectors revealed a per-patient pooled sensitivity of 95% (95%CI, 90-98%) and a specificity of 90% (95%CI, 87-93%) to detect ACS compared to invasive coronary angiography [50]. A second meta-analysis including 16 studies, totaling 1119 patients, found a sensitivity and specificity of 96% (95%CI, 93-98%) and 92% (95%CI, 89-94%) respectively [51]. 

The ROMICAT trial [30], which was an observational cohort study of patients with acute chest pain and normal initial ECG and troponins, found a sensitivity and NPV for the absence of plaques on CCT to detect ACS of 100%, and a sensitivity and NPV for non-significant CAD of 77% and 98% respectively. Furthermore, CCT results provided additional information beyond the TIMI score and traditional risk factors to predict ACS. [3]. Very recently, Chow *et al*. 52 reported a sensitivity of 98% (95%CI, 87-100%), a specificity of 100% (95%CI, 85-100%), a positive predictive value of 100% (95% CI, 90%-100%) and a NPV of 97% (95%CI, 80-100%) for 64-slice CCT compared to invasive coronary angiography in 107 patients with acute chest pain. 

The safety of CCT for early triage of low and intermediate-risk patients evaluated in the ED for acute chest pain has been addressed in several studies. In a prospective study with 103 patients presenting with acute chest pain to the ED who underwent CCT immediately before hospital admission, the absence of significant CAD was a good predictor of the absence of ACS during hospitalization and 5-month follow-up (NPV of 100%) [3]. Rubinstein *et al*. [53] reported higher predictive values of CCT to diagnose ACS compared to standard diagnostic criteria in the triage of acute chest pain patients and none of the patients with a normal CCT died or had a myocardial infarction over a 15-month period. Hollander *et al*. [54] prospectively evaluated 481 patients with a TIMI score <2 who presented to the ED with acute chest pain and had a negative CCT, recording only one death (0.2%; 95% confidence interval [CI] = 0.01% to 1.15%) of unclear etiology, no acute myocardial infarction and no revascularization procedures at one-year follow-up. Similarly, in a recent study including 70 low-to-moderate risk patients with chest pain who underwent CCT in the ED with negative results (<50% stenosis), none of the patients reported an adverse cardiac event over the 12-month follow-up [55].

Finally, the efficiency of CCT in the triage of patients presenting with acute chest pain in the ED has been the objective of several studies. In a preliminary study Hoffmann *et al*. [56] evaluated 40 patients with acute chest pain, normal cardiac biomarkers and a non-diagnostic ECG who underwent CCT in addition to the standard-of-care diagnostic evaluation prior to hospital admission. They found at least one significant coronary stenosis on CCT in all patients with final diagnosis of ACS whereas significant CAD was excluded in 26 of the 35 patients without a final diagnosis of ACS, suggesting that CCT could significantly decrease the number of patients who would require admission for a full ACS evaluation. In another study by the same group, low-risk patients were randomized to CCT or standard-of-care diagnostic evaluation, finding similar accuracy and safety for CCT but shorter time to diagnosis, potentially reducing costs [57]. In a study of 268 acute chest pain patients randomized to either immediate 64-slice CCT or conventional diagnostic strategy, CCT approach showed decreased hospital length of stay and hospital admissions [58]. In a study conducted by May *et al*. [59] in low-risk patients with acute chest pain, ED discharge based on negative CCT resulted in significantly shorter length of stay and lower hospital charges compared with the standard of care. The Computed Tomographic Angiography for the Systematic Triage of Acute Chest Pain Patients to Treatment (CT-STAT) trial that involved 750 patients at 15 centers across the United States presented in the American Heart Association Scientific Sessions 2009 found that costs were significantly reduced with CCT as compared to the standard of care [60]. 

More controversy exists regarding the application of the called “triple rule out” protocol using CCT. Extending the field of view to the entire thorax, and therefore imaging the aorta and pulmonary arteries besides the coronary arteries, CCT provides an attractive modality for ruling out three of the most life-threatening causes of acute chest pain: CAD, aortic dissection, and pulmonary embolism. However, a major technical challenge of this protocol is to obtain good contrast opacification in all three vascular beds. In the study conducted by Vrachiliotis *et al*. [61], a tri-phasic injection protocol with caudal-cranial scan acquisition resulted in consistent good opacification (>250 Hounsfield Units (HU) of the left main coronary artery, aorta and main pulmonary artery. However, it should be noted that the incidence of pulmonary embolism or aortic dissection is much lower compared to ACS, the quality to evaluate coronary arteries may be reduced, and the radiation dose needed is approximately 30-50% higher than for coronary studies alone. For these reasons, this protocol is usually recommended only if at least two out of the three diagnoses are reasonably suspected. Recently, high-pitch dual spiral technique using dual source CCT appears as an alternative to reduce radiation in cases where triple-rule-out protocol is deemed necessary (mean dose of 4.08 +/- 0.81 mSv) [62].

### Clinical Application of CCT in the ED for Chest Pain Evaluation: Selection of Patients

Appropriate patient selection is critical to avoid CCT overuse and to obtain clinically relevant information. As has been previously described, in the American College of Cardiology, The American Heart Association and American societies of cardiac imaging consensus, CCT is considered appropriate in patients with low to intermediate cardiac risk presenting with acute chest pain whose first set of cardiac biomarkers measurement are negative and who have no signs of acute myocardial ischemia on ECG [43]. Conversely, CCT is not recommended in patients with a high-risk profile (positive cardiac biomarkers or acute ECG changes) who should be managed according to the standard of care at each institution in accordance with the clinical practice guidelines. Triple rule out protocols should be performed only in cases where there is reasonable suspicion of at least two potentially lethal etiologies for the acute chest pain (ACS, aortic dissection and pulmonary embolism).

Patients with general contraindications to contrast-enhanced CT, such as history of a severe allergic reaction to an iodinated contrast material or impaired renal function (in general creatinine level >1.5 or estimated glomerular filtration rate <50%) should be excluded. Also, iodine contrast can be detrimental in patients with multiple myeloma and renal amyloidosis due to the risk of contrast-induced nephropathy and in patients with hyperthyroidism because contrast can trigger a thyroid storm. Common relative contraindications due to higher likelihood of poor-quality studies with most current scanners are body mass index greater than 40 kg/m2, atrial fibrillation or known preexisting coronary artery disease (previous coronary artery stent placement or bypass surgery). Additional risk factors, such as age, diabetes, hypertension, history of CAD and higher heart rate, have been reported to be independent predictors of poor-quality images in acute chest pain patients undergoing 64-slice CCT [63]. 

Patients should be trained to follow the breathing instructions before evaluation. Inability to hold their breath during acquisition significantly compromises image quality and may result in non-evaluable coronary artery segments. Additionally, to guarantee diagnostic image quality using CCT’s with less than 256 detectors, patients should ideally be in normal sinus rhythm and a heart rate<65 beats per minute during image acquisition. Patient’s heart rate should be measured during a breath-hold test previously to determine whether the administration of beta-blockers is necessary. If necessary, intravenous short-acting beta-blockers (eg, 5-20 mg of metoprolol) are administered before acquisition unless there is contraindication such as congestive heart failure, asthma or atrioventricular block. In addition, sublingual administration of nitroglycerin (0.4 mg) is usually administered before the scan to improve visualization of the coronary artery lumen. Nitroglycerin is contraindicated in individuals taking phosphodiesterase inhibitors, with increased intracranial pressure, severe aortic valve stenosis, severe obstructive hypertrophic cardiomyopathy, symptomatic hypotension or severe anemia. 

### CCT Protocol: Acquisition, Reconstruction and Post-Processing Analysis

A comprehensive description of the different aspects of the CCT protocol details for the evaluation of the coronary arteries is beyond the scope of the current review, however a brief explanation of the general phases is provided. After training the breathing instructions and optimizing the heart rate, the study can be initiated. With the patient in supine position, the exact location of the heart is identified obtaining antero-posterior topographic scan of the chest. Typically, the imaging volume should extend from 1-2 cm below the carina to the diaphragm. Contrast media (average of 70 ml) is injected intravenously at a flow rate of 4-5 ml/s. There are basically two techniques for contrast tracking to achieve good opacification of the coronary tree: bolus tracking and timing bolus techniques. The first one consists on the acquisition of a series of low-dose monitoring scans at the region of interest (usually at the level of the ascending or descending aorta) during contrast injection. When a predefined threshold inside the region of interest is reached (eg. 100 HU) the scan automatically begins. The timing bolus technique is based on prior estimation of circulation time using a small test bolus of contrast media followed by saline flush, injected at the same rate that will be used for the scan. Images can be acquired in helical mode or using prospective ECG-gating acquisition on non-helical mode if the heart rate is low and stable. A tube voltage of 120 kV, or 100 kV in thin patients (BMI <25 Kg/m2), is ordinarily employed. The tube current should be adjusted based on body mass index and modulation should be used if possible. Below 65 beats per minute, images are usually reconstructed using the half-scan technique, whereas at higher heart rates multi-segment reconstruction is used to decrease motion artifact. Optimal image quality is usually achieved in diastole. Images are usually reconstructed with a 0.75 mm section thickness and 0.4-mm overlap with 64-slice CCT. In order to reduce noise, images can be reconstructed with greater thickness. Isotropic resolution allows reformatting of imaging of any arbitrary plane without loss of image information. Different techniques as multiplanar reformation, curved multiplanar reformation, maximum intensity projection and volume rendering are used to evaluate the coronary arteries. 

### Detection of Obstructive CAD and Characterization of Coronary Atherosclerotic Plaque

Coronary artery stenoses are evaluated by visual analysis following the American Heart Association segmentation model. Once an atherosclerotic plaque is identified, the above-mentioned techniques are used to assess the degree of luminal stenosis by comparison with the healthy coronary segment immediately proximal to the lesion. The stenosis is usually graded as mild (<50% obstruction of luminal diameter), moderate (50-69%) or severe (≥70%) [64]. A significant stenosis is considered when the coronary luminal diameter is reduced by >50%. Currently, most available software packages provide semi-automatic evaluation of stenosis severity that has showed good agreement with quantitative analysis of invasive coronary angiography [65]. 

**Fig. (2):**
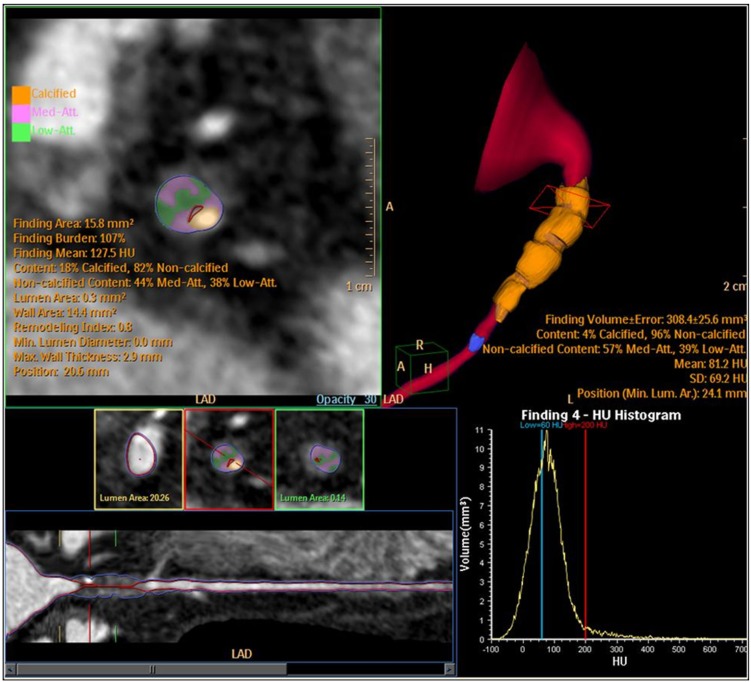
Example of semi-automatic quantification of coronary vessel stenosis and plaque characterization using dedicated software (Philips, Brillance 64). Calcified composition of the plaque is depicted in orange color and non-calcified plaque in green (low-attenuation) and in pink (middle-attenuation).

An interesting characteristic of CCT is the possibility of direct visualization of the vessel wall and therefore provides a good estimation of the atherosclerotic burden, which could help to better stratify patient’s risk. The goal of plaque characterization is to identify the vulnerable plaque, responsible for most ACS. Several studies have demonstrated good ability of CCT for plaque characterization compared to intravascular ultrasound [66, 67] or intravascular optical coherence tomography [68]. According to attenuation values in HU, plaques are usually classified as soft or lipid-rich (30-60 HU), fibrous (70-120 HU) or calcified (>350 HU) [69. Advanced software tools have been developed for this purpose and allow visualization and quantification of different components of the atherosclerotic plaque depicted in different colors (Fig. **2**). Furthermore, characterization of other features associated with plaque vulnerability, such as positive coronary remodeling is also feasible. In a study of 1059 patients who underwent CCT, authors found that those with plaques showing positive remodeling and low attenuation on CCT were at higher risk for ACS during 27-month mean follow-up compared to patients with plaques without these characteristics [70]. Another clinical demonstration was shown by Fernandez-Friera *et al*. [71] who described that plaque characteristics assessed by various imaging modalities, including CCT, intravascular ultrasound and near-infrared spectroscopy may predict clinical outcomes. 

### Evaluation of the Left Ventricle: Myocardial Infarction and Resting Wall Motion Abnormalities

Besides evaluation of the coronary arteries, CCT can be used to assess global and regional bi-ventricular function [72], myocardial perfusion [73, 74] and detection of intra-cavity thrombi. Left and right ventricular ejection fraction assessed by CCT has similar accuracy to cardiac magnetic resonance [75, 76]. Simpson’s or threshold-based volumetric methods are considered reliable to quantify ventricular ejection fraction. For Simpson’s method, endocardial contours at end-systole and end-diastole from the base to the apex of the heart are semi-automatically traced using dedicated software to derive volumes and ejection fraction. In the threshold-based 3D-volume measurement, the total chamber volume is calculated aggregating all the voxels that exceed a predefined attenuation threshold once the valve annulus level is selected. Volumes are displayed in different colors and manually corrected. Wall motion abnormalities can be visually analyzed playing a cine-loop of the cardiac phases in standard views of the heart (long and short axis orientation, two-, three- and four-chamber views). Finally, in addition to the assessment of cardiac and coronary anatomy, CCT provides valuable information about the lungs, mediastinum, chest wall and upper abdominal structures, which may also be responsible for the chest pain in these patients. 

### CCT in the ED: Advantages and Limitations

In summary, extensive evidence exists regarding the accuracy, safety and efficiency of CCT for the triage of acute chest pain patients. Currently, the use of CCT for the evaluation of low-intermediate cardiac risk patients presenting with acute chest pain in the ED is considered appropriate. Additionally, CCT allows plaque characterization and provides valuable information about systolic function, myocardial perfusion and extra-cardiac findings. Modern scanners such as 320-detectors and dual-source models, which allow image acquisition in one cardiac cycle without the need for additional medications or breath-hold, will further facilitate its application in this setting. 

Nevertheless, some limitations should be taken into account. First, CCT evaluation is associated with radiation exposure so conscious risk-benefit analysis should be performed for each individual case. However, by adjusting tube voltage and applying dose modulation or prospective ECG protocols, the average dose of radiation is smaller than that necessary to perform a diagnostic coronary angiography or a nuclear stress test. New scanners will allow further reduction in radiation dose. Second, the necessity of administering contrast media to visualize the coronary arteries is a relative contraindication in patients with impaired renal function. Several conditions such as morbid obesity, inability to follow the breathing instructions, atrial fibrillation, severe calcification of the coronary vessels or some types of coronary stents compromise image quality and make accurate coronary artery stenosis assessment. Again, recent technical advances have extended the capability of CCT to image morbidly obese patients or patients with arrhythmias with good image quality. Finally, as with conventional coronary angiography, CCT provides only anatomic information but no evidence of the physiological relevance of the lesion on coronary flow, which can be very important particularly in the assessment of intermediate stenosis. In these cases, functional tests may be useful to fully characterize the clinical impact of the lesion. Ongoing randomized studies will help to support and to answer other questions regarding the safety and cost-efficiency of CCT for the triage of patients with acute chest pain. 

## COST-EFFECTIVENESS 

The cost of ED evaluation and treatment of patients with chest pain places a significant financial burden on health care systems. Among the almost 6 million patients who present annually to United States ED’s because of acute chest pain, only approximately 20% of those evaluated with traditional testing for cardiac ischemia ultimately receive the diagnosis of coronary heart disease. [77], Because “ruling out” ACS requires time and advanced testing, it follows that as many as 80% of patients undergoing this evaluation may require admission or prolonged observation that will prove to be unnecessary at the end of the evaluation.. According to national statistics on community hospital stays in the United States [77, 78], in 2006 the number of discharges for non-specific chest pain was 856,948, with a mean length of stay of 1.8 days. That year the national bill for nonspecific chest pain amounted to more than $11.2 billion.

Recognized standards for the cost-effectiveness of any testing modality rely on an analysis that incorporates the tests effects on survival, quality of life and cost using a lifetime time horizon. Cost effectiveness in this setting is defined as the ratio of the direct and indirect costs for the test and the number of patients correctly diagnosed as having CAD [79]. A decrease in the cost per correct diagnosis thus indicates improved cost effectiveness. The cost of a diagnostic test strategy comprised the following components: direct cost (reimbursement rates for the modalities) multiplied by the number of patients and indirect cost (cost of subsequent tests, cost of complications associated with the diagnostic modality, cost of additional tests, and cost resulting from diagnosis of a patient as false negative, multiplied by the respective number of patients).

There have been several attempts to assess the cost-effectiveness of CAC score. O’Malley *et al*. [80] constructed a decision analytic model of the additional value of CAC score to the FRS. Any CAC score greater than 0 would increase the relative risk 4-fold. Shaw *et al*. [18] developed a similar decision-analytic model, finding that in individuals with estimated risk of coronary events below 0.6% per year, the incremental cost-effectiveness ratio approached $500 000. , When the estimated even rate increased the cost-effectiveness ratio decreased, meaning that the effectiveness of the examination significantly improved ($42 339 and $30 742 when the risk of events was1% and 2% per year, respectively). The results of various modeling approaches agree in their assessment that an appropriate use of CCT in low to intermediate risk patients with acute chest pain is associated with cost savings compared with stress testing, especially in younger men and women [78, 82, 83] . Ladapo *et al*. [82] developed a simulation model to compare the costs and health effects of CCT for acute chest pain evaluation with a standard-of-care algorithm that included measurement of cardiac markers for triage of patients to early discharge, stress testing, or invasive coronary angiography. According to the simulations, among men the incremental cost-effectiveness ratio for CCT was $6,400 per quality-adjusted life year; among women, CCT was cost-saving. The authors concluded that CCT– based triage of patients with low risk chest pain- is moderately more cost-effective than the standard of care, particularly for women, who traditionally present a greater diagnostic challenge in the evaluation of acute chest pain than men do.

## RADIATION EXPOSURE FOR CCT AND CAC SCORE SCAN

The volume of cardiac diagnostic procedures involving the use of ionizing radiation has increased rapidly in recent years. CCT volume doubled between 2002 and 2003, to 485,000 cases [84] and has continued to grow since then. CCT, SPECT, and invasive angiography all expose patients to radiation. Mean effective dose for calcium score using retrospective gating ranges from 1.0 to 6.2 mSv, depending on the protocol and scanner used. Mean effective dose is lower using prospective gating, with a range from 0.5 to 1.8 mSv, although this does not include any 64-slice studies. Mean effective dose for CCT ranges from 4.0 to 21.4 mSv [85]. Radiation doses of retrospectively gated 64-slice CT typically range from 7 to 14 mSv when dose modulation strategies are used [86]. This exposure is comparable to stress SPECT (9 to 12 mSv) and lower than a thallium myocardial perfusion scan (18 to 21 mSv) [87, 88] but higher than the effective radiation dose from an invasive coronary angiography (5 to 7 mSv). 

Model-based calculations suggest that lifetime cancer risk from standard CCT scans varies from 1 in 143 (0.7%) for a 20-year-old woman to 1 in 3261 for an 80-year-old man (<0.03% ) [86]. In comparison, US women have a 1 in 8 (12.5%) lifetime chance of developing invasive breast cancer, and the overall cancer risk for 75-year-old men/women is 6%. Thus, even if the model-based assumptions of the CCT–based cancer risk estimates are valid, the incremental risk seems low but negligible [30]. Moreover, advances using prospective ECG gating indicate that a low radiation scan option (<5 mSv) may be increasingly feasible to image selected populations of ED patients [89, 90].

Procedures that utilize ionizing radiation should be performed in accordance with the As Low As Reasonably Achievable (ALARA) philosophy. A number of techniques can be used to minimize radiation dose:

- For CAC, prospective gating is recommended. Ideally, the non-contrast calcium score scan should be examined before proceeding with CT angiography because widespread calcification may render many coronary segments difficult to interpret.
- For CCT, ECG-controlled tube current modulation should be employed when it is expected that multiple reconstructions at different portions of the cardiac cycle will not be necessary to interpret the images 91].

Another component of dose reduction is minimization of CCT scan length by using the scout and, when available, the calcium score scan. One approach considered by Hausleiter *et al* [92] is reducing tube voltage from the standard 120 kV to 100 kV because dose varies approximately with the voltage squared. 

Multiple sources enable increasing the pitch of a scan, ie, less overlap between gantry rotations, and correspondingly a lower dose. Another possibility for lowering the dose in CCT is the employment of prospective gating to only acquire images during diastole , combined with “step-and-shoot” non-spiral scanning [93] or longer detector arrays (eg, 256 detectors) enabling non-spiral whole organ imaging. The sensitivity, specificity, and dosimetry of such strategies remain to be established, but this technology is advancing rapidly, and multiple CT scanner manufacturers have recently announced the release of step-and-shoot algorithms.

## CONCLUSIONS

In summary, both CAC score and CCT are cost-effective imaging tests that help to identify patients presenting with chest pain to the ED who may be safely discharged. The negative predictive utility of both tests largely depends on the pre-test probability of disease in the population studied. Until additional evidence is obtained from larger multi-center studies, current evidence and expert opinions suggest that:

1. A zero-calcium score has a very good negative predictive value if obtained in a middle age or older patient at low or intermediate risk, but false negatives may occur in high-risk and younger patients who may have a non-calcified plaque as a culprit lesion prior to the development of coronary calcification. In these cases, a follow up CCT angiogram is recommended.
2. A non-zero CAC does not necessarily imply the presence of coronary stenosis. Therefore, in patients with low-intermediate probability of ACS, a non-zero CAC should also be followed by a CCT angiogram or a functional test, whereas in those at higher risk, invasive coronary angiography should be considered.

## Figures and Tables

**Table 1.** Thrombosis in Myocardial Infarction (TIMI) Risk Score

Risk Factors
- Age > 65 years
- History of known coronary artery disease (>50%)
- Severe angina symptoms (>2 episodes of chest pain in the last 24 hours)
- ST-segment deviation on admission ECG (persistent depression or transient elevation)
- Elevated serum cardiac biomarkers (troponins)
- Use of aspirin in the last 7 days before presentation
- 3 or more cardiac risk factors (age, male sex, family history, hyperlipidemia, diabetes, smoking, obesity)

Low risk= 0-2 points; Intermediate risk= 3-4 points; High risk= 5-7 points. Each positive factor is worth it one point.

**Table 2.** Summary of the Most Representative Published Studies Examining the Accuracy of Coronary Artery Calcification to Detect Significant Coronary Artery Disease in an Emergency Department Population

Year	Scanner	n	Subjects	Ss (%)	Sp (%)	PPV (%)	NPV (%)
McLaughlin ^24^1999	EBCT	134	53±2y 40%male	100	54	15	98
Laudon ^25^1999	EBCT	105	48±5y 54%male	100	63	30	100
Georgiou ^28^2001	EBCT	192	53±10y 54%male	97	55	26	97
Sarwar* ^26^2009	EBCT/ MDCT	10355	18studies	98	40	68	93
Laudon ^31^2010	EBCT	263	45±7y 60%male	97	57	23	99

Ss= Sensitivity; Sp= Specificity. PPV= Positive predictive value to diagnose significant coronary artery disease; NPV= Negative predictive value to rule out significant coronary artery disease;

* Systematic review of the accuracy of coronary artery calcium to predict the presence or absence of significant coronary artery stenosis by invasive coronary angiogram in symptomatic patients.

**Table 3.** Summary of the Most Representative Published Studies on Cardiac Computed Tomography in Patients Presenting with Acute Chest Pain to the ED

Author	Year	Scanner	n	Subjects’ TIMI & age	Ss (%)	Sp (%)	PPV (%)	NPV (%)
Sato [48]	2005	6-(N=26) & 16-slice (N=4)	31	TIMI≤2 58±14y (ACS) 50±13y(non ACS)	95.5	88.9	95.5	88.9
Gallagher [94]	2006	64-slice	85	TIMI= 0.8±0.8 49±11y	86%	90%	99%	50%
Vanhoenacker [50] (meta-analysis)	2007	4-(1study), 16-(2 studies), 32- (1 study) & 64-slice (4 studies)	9 studies; 566 subjects		95%	90%	ND	ND
Hoffmann [30] (ROMICAT)	2009	368	64-slice	53±12y	77%	87%	37%	98%
Athappan [51] (meta-analysis)	2010	4-(2 studies), 16-(6 studies), 40-(1 study) & 64-(10 studies)	16 studies; 1119 subjects		0.96	0.92	ND	ND
Chow [52]	2010	64-slice	107	54±10y	98*	100*	100*	97*

ND= No data; Ss= Sensitivity; Sp=Specificity; PPV= Positive predictive value to diagnose acute coronary syndrome; NPV= Negative predictive value to rule out acute coronary syndrome

* Significant coronary artery disease instead of acute coronary syndrome as outcome.

## ABBREVIATIONS

EBCT: = Electro-beam computed tomography
ED: = Emergency department
ECG: = Electrocardiogram
HU: = Hounsfield Unit
ACS: = Acute coronary syndrome
CAD: = Coronary artery disease
CAC: = Coronary artery calcium
CCT: = Coronary computed tomography
SPECT: = Single-photon emission computed tomography
NPV: = Negative predictive value
